# Comparative Analysis of p*K*_a_ Predictions for Arsonic Acids Using Density Functional Theory-Based and Machine Learning Approaches

**DOI:** 10.1021/acsomega.4c10413

**Published:** 2025-01-16

**Authors:** Miroslava Nedyalkova, Diana Heredia, Joaquín Barroso-Flores, Marco Lattuada

**Affiliations:** ‡ Swiss National Center for Competence in Research (NCCR) Bio-inspired Materials, University of Fribourg, Chemin des Verdiers 4, Fribourg CH-1700, Switzerland; § Department of Chemistry, University of Fribourg, Chemin du Musée 9, Fribourg 1700, Switzerland; ± Department of Inorganic Chemistry, Faculty of Chemistry and Pharmacy, University of Sofia ‘St. Kl. Ohridski’, Sofia 1504, Bulgaria; ∥ School of Chemical Sciences and Engineering, Yachay Tech University, Urcuquí 100119, Ecuador; † Centro Conjunto de Investigación en Química Sustentable UAEM-UNAM, Carretera Toluca-Atlacomulco Km 14.5, Unidad San Cayetano, Toluca, Estado de México 50200, México; ¶ Instituto de Química, Universidad Nacional Autónoma de México. Circuito Exterior S/N Ciudad Universitaria, Alcaldía Coyoacán, Ciudad de México CP 05410, México

## Abstract

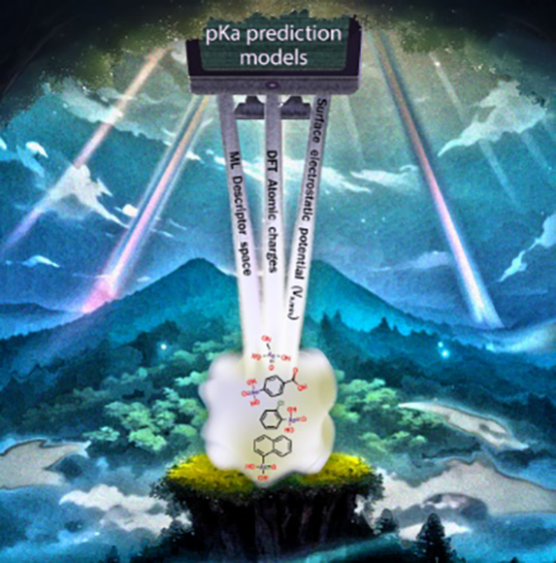

Arsonic acids (RAsO(OH)_2_), prevalent in contaminated food, water, air, and soil, pose significant environmental and health risks due to their variable ionization states, which influence key properties such as lipophilicity, solubility, and membrane permeability. Accurate p*K*_a_ prediction for these compounds is critical yet challenging, as existing models often exhibit limitations across diverse chemical spaces. This study presents a comparative analysis of p*K*_a_ predictions for arsonic acids using a support vector machine-based machine learning (ML) approach and three density functional theory (DFT)-based models. The DFT models evaluated include correlations to the maximum surface electrostatic potential (*V*_S,max_), atomic charges derived from a solvation model (solvation model based on density), and a scaled solvent-accessible surface method. Results indicate that the scaled solvent-accessible surface approach yielded high mean unsigned errors, rendering it less effective. In contrast, the atomic charge-based method on the conjugated arsonate base provided the most accurate predictions. The ML-based approach demonstrated strong predictive performance, suggesting its potential utility in broader chemical spaces. The obtained values for p*K*_a_ from *V*_S,max_ show a weak prediction level, because the way of predicting p*K*_a_ is related only to the electrostatic character of the molecule. However, p*K*_a_ is influenced by many factors, including the molecular structure, solvation, resonance, inductive effects, and local atomic environments. *V*_S,max_ cannot fully capture these different interactions, as it gives a simplistic view of the overall molecular potential field.

## Introduction

The toxicity and contamination potential of arsonic acids are influenced by their p*K*_a_ values, which determine the relative concentrations of neutral and ionized species in the environment. The p*K*_a_, or acid dissociation constant, is a critical parameter that affects how these compounds behave under various pH conditions, impacting their mobility, bioavailability, and environmental persistence.^1^ In aqueous environments, pH affects whether these arsonic compounds exist in their neutral or ionized forms. For arsonic acids, the ionized and nonionized forms have different chemical reactivity and biological interactions. For example, arsenate ions (As(V)) and arsenite ions (As(III)) exhibit varying toxicological profiles, and their presence is directly influenced by the pH and p*K*_a_ values.

The toxicological behavior of arsonic acids is closely tied to their ionization state, which depends on p*K*_a_. Accurately predicting p*K*_a_ values helps in identifying conditions under which the more toxic forms might dominate, thereby assessing the potential health risks to aquatic organisms and humans. Arsonic acids’ mobility in water is largely dependent on their charge state. Compounds that are predominantly neutral can diffuse more easily through soil and water compared to their charged counterparts. For instance, at higher pH levels, arsonic acids may ionize into negatively charged species, reducing their adsorption to negatively charged soil particles and increasing their mobility in water bodies. This increased mobility raises concerns about contamination of groundwater and the bioaccumulation of toxic arsenic species in plants and aquatic life. The charge state of arsonic acids, influenced by their p*K*_a_, affects how they interact with soil and sediments. Negatively charged species are less likely to absorb negatively charged soil particles, leading to higher mobility in water. Understanding p*K*_a_ values aids in predicting these adsorption behaviors, which is crucial for environmental fate modeling and designing remediation strategies

Knowing p*K*_a_ helps in predicting the predominant species under specific environmental conditions, such as acidic or alkaline settings, enabling the design of more effective remediation strategies.^2−4^ This is particularly relevant, as some of these compounds degrade into more toxic inorganic arsenic forms through natural processes. For instance, in the natural pH range of 5.5–8.5, arsenite is predominantly present in its protonated, more toxic form. However, as pH decreases, arsonic acid derivatives deprotonate, acquiring a net electrostatic charge, reducing its toxicity and enhancing removal efficiency.^1,5,6^ The p*K*_a_ values for arsonic acids like benzoic acid, *p*-chloroaniline, 2-chlorophenol, 2,4-dichlorophenol, 2,4,5-trichlorophenol, and 2,4,6-trichlorophenol fall within the range of environmentally significant pH conditions from 6.5 to 9.5, where the dominant arsenic species will be a mixture of H_2_AsO_4_^–^ and HAsO_4_^2–^, meaning that mixed systems will prevail under such conditions, with both species contributing to total absorption. The charge state of arsonic acids affects their adsorption into soil particles. Generally, negatively charged species (at higher pH) are less likely to absorb negatively charged soil particles, increasing their mobility and bioavailability. In contrast, neutral species are more likely to passively diffuse through cell membranes of microorganisms and plant roots compared with charged species. This affects the uptake and bioaccumulation of arsonic acids.^7^ The speciation of arsonic acids also affects their uptake by plants. For example, arsenates (As(V)) are taken up by phosphate transporters, while arsenites (As(III)) are more likely to be taken up by aquaglyceroporins.^8^ Different microbial species have evolved specific transport systems for various arsenic species. The ionic state of the arsonic acid, determined by its p*K*_a_ and environmental pH, influences which uptake mechanism is the most effective.^9^

Phenylarsonic acid compounds have been a primary component of animal feed additives for several decades, primarily used to promote growth and control bacterial and parasitic diseases in livestock. Nitarsone and roxarsone (molecules **24** and **26** in the present study, respectively) are widely used as organo-arsenic-based bird feed additives, which are then excreted as inorganic arsenic, causing a steep rise in the soil concentration of arsenic, which can in turn also be transported into groundwater.^10−12^ These compounds undergo minimal metabolism in animal bodies and are largely expelled through manure and urine. Despite the low toxicity of these drugs, they can decompose into more harmful inorganic arsenic forms, specifically arsenite and arsenate, through both biotic and abiotic processes. Therefore, p*K*_a_ calculations are essential for predicting and evaluating the environmental toxicity of different types and sources for such a contaminant. The motivation of developing a robust model for p*K*_a_ predictions stems from the fact that p*K*_a_ is not only important for the environmental contex but also is the key descriptor when we want to design catalytic systems^4,13,14^ and screen drug-like molecules for optimal pharmacokinetics.^15^

Indeed, developing accurate and efficient prediction models for p*K*_a_ is complex due to the multifaceted influences on ionization behavior. Each of the factors, such as the conformational flexibility of the ionizable groups, structural symmetry, unusual heterocyclic structures, multiple ionization centers, charge transfer in conjugated systems, tautomerism, and intra- and intermolecular interactions, affects the level of prediction.

Various computational methods have been developed for calculating p*K*_a_ values, mainly divided into two major categories: macroscopic and microscopic. For small molecules, the standard approach involves calculating the free energy difference between protonated and deprotonated species within a thermodynamic cycle, where solute–solvent interactions are represented via an implicit solvation model that approximates the electrostatic potential.^16−18^ On the other hand, a two-step procedure can be used to capture the electrostatic solvation effect. First, the restricted electrostatic potential generates a point charge distribution.^19^ This is followed by solving the Poisson equation to obtain solvation energies.^20,21^ When combined with conformational sampling, this approach is particularly suitable for large systems, such as proteins with multiple protonation sites.^22,23^ While many protocols reproduce experimental p*K*_a_ values with reasonable accuracy, performance varies with system type, especially in large, flexible, or highly charged species, which challenges underlying assumptions. Numerous correction schemes and tailored protocols address these limitations, although a comprehensive review exceeds the scope here; selected references provide further insight.^24−32^

The simplest way to estimate the p*K*_a_ of an acid in solution using quantum methods is based on the calculation of the equilibrium reaction Gibbs energy: HA _(soln)_ ⇌ A_(soln)_^–^ + H_(soln)_^+^.^33−35^ One of the most difficult aspects to estimate theoretically is the free energy of the solvated proton.^36^ The challenge in accurately predicting the solvation free energy of ionized species, particularly hydrogen ions (H^+^), stems from inherent limitations in current solvation models, as highlighted in the solvation model based on density (SMD) literature.

Several studies have attempted to achieve reliable estimates for Δ*G*_solv_(H^+^) by either using experimental data or theoretical approaches, which generally place the value between −252.6 and −271.7 kcal/mol.^37−40^ Currently, the most widely accepted value is −265.6 ± 1 kcal/mol. Unlike neutral molecules, which tend to yield relatively precise hydration free energies, ionic species exhibit significantly larger prediction errors. For cationic species, errors are reported to be around 2–3 times higher than those for neutral molecules, while anionic species show discrepancies ranging from 3 to 6 times greater. These variations reveal the complexity of accurately capturing the solvation environment for ions largely because the electrostatic interactions in solution introduce challenges that are not as prominent in neutral solvation. To address these discrepancies, various strategies have been developed. One commonly employed approach is scaling the solvent-accessible surface area (SASA) model to better represent the solvation of the ions. Another common method involves introducing explicit water molecules to more accurately capture the solvation environment of the ions. Additionally, another method is to employ calibration curves to refine the predicted solution free energies. Calibration curves work by adjusting theoretical solvation energies against experimental data, effectively creating a tailored correction for the discrepancies inherent in current solvation models. This method is especially beneficial in addressing the larger errors observed for charged species, where traditional models such as SMD often fall short.

The application of thermodynamic cycles for minimizing errors in p*K*_a_ calculations by combining gas-phase and solution-phase deprotonation reactions is one very common approach. In the work of Ho,^31^ regarding the necessity of thermodynamic cycles for continuum solvent models, direct solvation models can provide competitive accuracy for calculating p*K*_a_'s and reduction potentials. Casasnovas et al.^41^ build on this concept by examining alternative protocols for p*K*_a_ prediction that avoid traditional gas-phase calculations, suggesting that continuum solvent models can offer accurate predictions without fully relying on thermodynamic cycles. Sutton et al.^42^ assess the impact of thermodynamic cycles and explicit solvation on p*K*_a_ calculations for carboxylic acids, revealing that the SMD solvation model and inclusion of explicit solvent molecules enhance accuracy in complex environments. Dissanayake and Senthilnithy^43^ focus on hydroxamic acids, showing that ab initio thermodynamic cycles accurately capture multiple deprotonation sites and complex acid structures. Pliego^44^ critiques standard thermodynamic cycle methods for p*K*_a_ prediction, highlighting limitations in solvation models and proposing refinements to improve reliability. Despite these complexities, the thermodynamic cycle approach generally performs well for compounds with a low or moderate structural complexity. However, for flexible molecules, where conformational changes between the gas and solution phases can be significant, the standard thermodynamic cycle approach becomes less effective. The main limitation for the thermodynamic cycle for p*K*_a_ involves calculating the free energy of deprotonation in the gas phase, where the proton is removed from the molecule, creating a conjugate base. The same deprotonation reaction is then considered in solution using solvation models to account for the energy change due to solvent interactions.

Due to limitations in thermodynamic cycles, the SMD model offers a more computationally efficient and versatile approach for quick calculations across solvents and inclusion of nonelectrostatic interactions, but it lacks the detailed solute–solvent interaction modeling of thermodynamic cycles and may struggle with explicit proton solvation. A continuum solvation model based on the quantum mechanical charge density of a solute molecule interacting with an SMD was used by Sabuzi et al.^45^ for accurately predicting p*K*_a_ for carboxylic acid derivatives using B3LYP and CAM-B3LYP. As a result of these findings, it was demonstrated that neither complex theory nor external factors are necessary for accurate prediction of carboxylic acid p*K*_a_. Coote et al.^46^ have demonstrated that thermocycle-based approaches for p*K*_a_ prediction have limitations when dealing with complex organic molecules where all molecular conformations must be considered.

The computational costs associated with thermodynamic cycle-based approaches for predicting p*K*_a_ values can be significant. These costs arise due to the complexity of the calculations necessary to determine a chemical reaction’s solution-phase Gibbs free energy, which involves multiple steps, including two geometry optimizations and determining the change in Gibbs free energy, Δ*G*. This process can be computationally intensive, making them unattractive for systematic conformational searches.

As we see, the available literature theoretical equation for calculating p*K*_a_ based on a proton transfer reaction between an acid and a single water molecule has been derived using the general chemical equilibrium relationship. The derived equation was then compared with two recently proposed equations that utilize thermodynamic cycles but yield different p*K*_a_ predictions. The analysis revealed that one of these thermodynamic cycles is incorrect, and its seemingly better performance is attributed to an erroneous solvation free energy value for the H_3_O^+^ ion. Furthermore, the investigation highlighted inconsistencies in the parametrization of the PCM-UAHF solvation model.

The study was based on prediction of the p*K*_a_ values for carboxylic compounds by calculating *V*_S,max_ values over the acidic hydrogen atoms. The *V*_S,max_ values over acidic hydrogen atoms represent the maximum electrostatic potential on the molecular surface at or near these hydrogen atoms, specifically in molecules with acidic groups, such as carboxylic acids. This metric helps identify the degree to which these hydrogen atoms are likely to donate a proton (H^+^) to a base, which is directly related to the molecule’s acidity. In practical terms, a higher *V*_S,max_ over an acidic hydrogen atom usually corresponds to stronger acidity (a lower p*K*_a_ value) because it reflects a greater electron deficiency at that site. This makes hydrogen more likely to dissociate as a proton. Therefore, by calculating *V*_S,max_ values over acidic hydrogens in various compounds, researchers can accurately predict and compare their p*K*_a_ values, providing insights into their acid strength without requiring experimental measurements. Similarly, calculating *V*_S,min_ over basic nitrogen atoms allows us to predict p*K*_b_ values for amines, and when used in conjunction with the previous model, isoelectric point values can be accurately predicted for amino acids. Calculated atomic charges and experimental p*K*_a_ values of carboxylate fragments in their anionic form showed a good correlation.^47,48^ The applied support vector machine (SVM) model was used to predict p*K*_a_ values; the prediction rate was lower than that of DFT but better than that of electrostatic potential surface (ESP). To the best of our knowledge, no systematic study has used the DFT, ML, and ESP approaches to calculate p*K*_a_ values for arsonic acids.

A useful tool for studying the charge distribution around the molecule is calculating the EPS. On the EPS, areas with high electron density have minimum values, the lowest of which, on the confinements of a given atom, are called *V*_S,min_. These minimum values mean that over these regions, the electrons pass more time on average, and as the electrons have a negative charge, this results in minimum values of EPS. On the contrary, the regions with low electron density have maximum values, and the larger homologous electrostatic values are called *V*_S,max_.

In the context of an acid–base reaction, the ease with which the proton is released from the corresponding acid molecule is important. As the bond maintains the atoms joined in a molecule, the weaker the bond, the easier the proton releases and, therefore, the more acidic the molecule is. From these facts, the relationship between the EPS value over acidic hydrogens and the p*K*_a_ value of arsonic acids is studied.

In recent years, machine learning techniques have been applied to many scientific topics, including prediction of p*K*_a_ values. Cai and co-workers reported a deep learning-based p*K*_a_ predictor, DeepKa, trained on data generated by constant-pH simulations.^49^ Reis and co-workers also reported a deep learning-based p*K*_a_ predictor, pKaI, which was trained on p*K*_a_ values calculated by a continuum electrostatics method.^50^ Another protein p*K*_a_ prediction paper from Gokcan and Isayev introduced a new empirical scheme based on deep representation learning trained on experimental p*K*_a_ data.^51^ The advantages of support vector machine and cascade deep forest are that they could perform well on small data sets.^52,53^ This is the reason why we use the SVM for our case. To gain physical insights from the ML models, we evaluated feature importance and determined the features causing p*K*_a_ shifts for the explored set of 35 arsonic acids.

This study reports p*K*_a_ calculations for 35 arsonic acid derivatives using one ML-based method and three DFT-based approaches. These methods include a direct thermodynamic cycle calculation with a solvent-accessible surface-corrected solvation model (SMD-SAS), a multivariate regression analysis based on atomic charges of the carboxylate, and a linear correlation of the maximum surface potential (*V*_S,max_) on the acidic hydrogen atom. The results of these methods are compared, highlighting their relative accuracy and applicability to predicting p*K*_a_ values for arsonic acids. This set encompasses a wide range of derivatives including 1-naphthylarsonic, 2,4-dimethoxyphenylarsonic, and a variety of phenylarsonic acids substituted with different functional groups such as amino, chloro, methoxy, methyl, nitro, and hydroxy groups. Electron-donating groups such as methoxy and methyl tend to increase the electron density on the arsonic acid moiety, potentially leading to higher p*K*_a_ values, suggesting weaker acidic strength. Conversely, electron-withdrawing groups like nitro and chloro are expected to lower the p*K*_a_, indicating stronger acids. The position of substituents on the aromatic ring (ortho, meta, and para) relative to the arsonic group significantly affects the p*K*_a_ due to differences in electronic and steric interactions. For instance, ortho-substituents might cause steric hindrance that could influence the accessibility of the arsonic acid for proton dissociation. Bulkier groups such as *n*-butyl and hexyl introduce steric strain that could impact the overall geometry of the molecule, potentially affecting the ease with which the acid can dissociate. Among the notable compounds, 3-acetylamino-4-hydroxyphenylarsonic illustrates a complex interplay of electron-donating (amino) and electron-withdrawing (acetyl) effects, which may result in a balanced p*K*_a_ value that could be critical for specific biochemical or environmental interactions. Similarly, 4-nitronaphthalen-1-yl-1-arsonic acid, with a strong electron-withdrawing nitro group adjacent to the aromatic ring, is one of the strongest acids in the set.

This detailed examination not only guides the synthesis and application of these compounds in various fields, such as medicinal chemistry and environmental science, but also sets the stage for further experimental or computational studies to precisely quantify how these structural elements affect the p*K*_a_ values of arsonic acids.

The study was leveraging p*K*_a_ prediction models not just as a theoretical exercise but as a practical tool that informs the synthesis, application, and management of arsonic acids. We conduct experiments based on one data set and application of four computational and ML approaches to achieve the best overall performance compared with the experimental values. In this manner, we achieve state-of-the-art results across four distinct types of applications of different approaches to goal-directed tasks to the target properties.

## Materials and Methods

In this study, we explored the accuracy of various methods to predict the p*K*_a_ values of arsonic acids by focusing on electrostatic and solvation descriptors, including *V*_S,max_, and the scaled solvent-accessible surface (sSAS). *V*_S,max_ is an approach that quantifies the maximum electrostatic potential on the surface of acidic hydrogen atoms and was chosen to capture the interaction potential between molecules and aqueous environments, making it a crucial predictor of p*K*_a_. This approach provides insight into electronic properties that influence acidity, essential for modeling p*K*_a_ values accurately. Additionally, the sSAS method was implemented to better represent the solute–solvent interface, capturing solvation effects that significantly impact acidity by adjusting the solvent-accessible surface based on the solvent radius. The sSAS correction is supposed to enhance the predictive power of the model.

**Figure 1:**
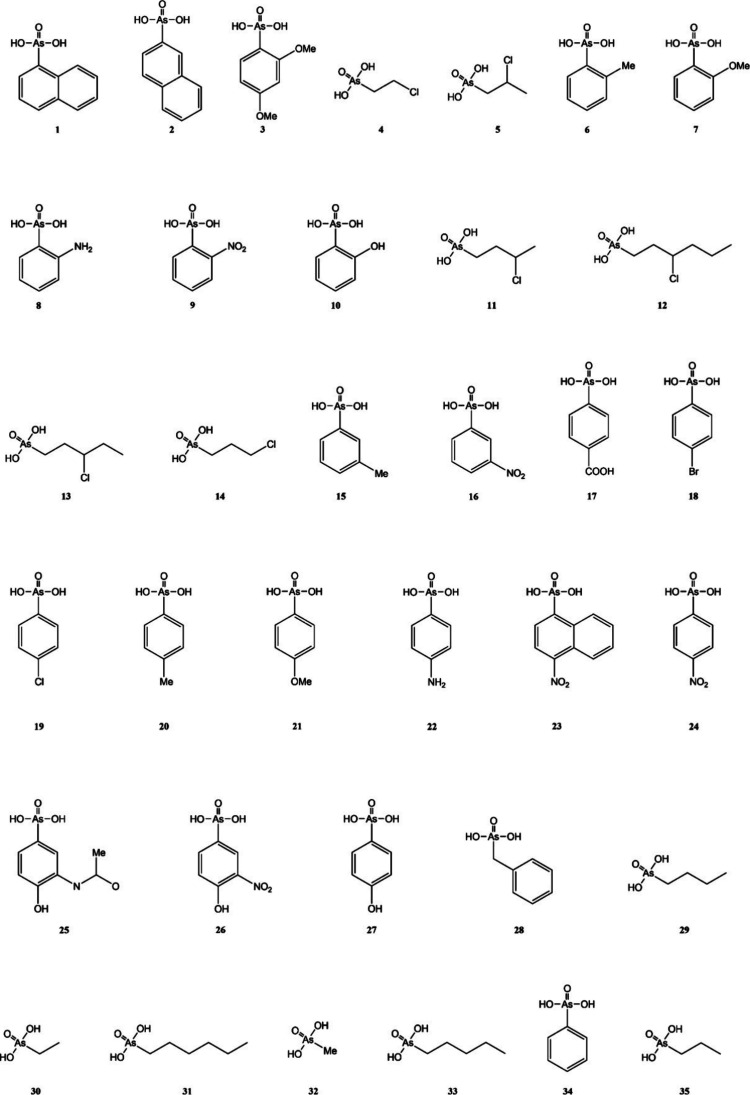
Chemical structures of the explored arsonic acids.

To calculate *V*_S,max_ values, the structures of the 35 arsonic acids in Figure 1 were optimized at the ωB97XD/cc-pvTZ level of theory using the Gaussian suite of programs.^54^ To corroborate that the optimized structures corresponded to a minimum, all vibrational frequencies were calculated too, and no negative frequencies were found. Their corresponding WFX files (we are using the WFX file to store wave function data from quantum chemistry calculations; the information within a WFX file includes essential details about the wave function, such as electron density, orbital shapes, and atomic coordinates, all of which are crucial for analyzing molecular properties and electronic distributions) were then processed with MultiWFN^55^ to calculate the electrostatic potential values around the molecules, thus obtaining *V*_S,max_ values for relevant atoms (vide infra), i.e., acidic hydrogen atoms in our case.

MultiWFN uses eqs 1 and 2to quantify both  and , which correspond to the average of positive and negative ESPs over the van der Waals surface,^36^ respectively:
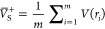
1
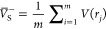
2where *i* and *j* are indices of the positive and negative regions of sampling points over the EPS. The arsonic acid group has two acidic hydrogen atoms and therefore also has two p*K*_a_ values corresponding to the first and second deprotonation events. These two values are obtained from experimental measurements reported previously, and they are collected in Table 1 in which they are labeled as p*K*_a1_ for the first deprotonation and p*K*_a2_ for the second deprotonation.

**Table 1:** *V*_S,max1_ (eV), *V*_S,max2_ (eV), and Experimental p*K*_a1_ and p*K*_a2_ Values for All 35 Arsonic Acid Derivatives Used in This Study

no.	**compound name**	*V*_S,max1_ (eV)	*V*_S,max2_ (eV)	p*K*_a__1_	p*K*_a__2_
1	1-naphthyl arsonic acid	2.13	2.10	3.66	8.66
2	2-naphthyl arsonic acid	2.20	2.07	4.2	8.46
3	2,4-dimetoxyphenyl arsonic acid	1.86	1.86	4.35	9.55
4	2-chloroethyl arsonic acid	2.38	2.36	3.68	8.37
5	2-chloropropyl arsonic acid	2.28	2.06	3.76	8.39
6	2-methylphenyl arsonic acid	2.11	2.11	3.82	8.85
7	2-methoxyphenyl arsonic acid	1.94	1.94	4.08	9.40
8	2-aminophenyl arsonic acid	2.32	2.04	3.79	8.93
9	2-nitrophenyl arsonic acid	2.13	2.12	3.37	8.54
10	2-hydroxyphenyl arsonic acid	1.97	1.97	4.00	7.92
11	3-chlorobutyl arsonic acid	2.20	2.19	3.95	8.85
12	3-chlorohexyl-1-arsonic acid	2.20	2.17	3.51	8.31
13	3-chloropentyl-1-arsonic acid	2.20	2.17	3.71	8.77
14	3-chloropropyl arsonic acid	2.29	2.25	3.63	8.53
15	3-methylphenyl arsonic acid	2.17	2.06	3.82	8.60
16	3-nitrophenyl arsonic acid	2.50	2.39	3.41	7.80
17	4-arsenobenzoic acid	2.34	2.23	4.22	8.44
18	4-bromophenyl arsonic acid	2.34	2.22	3.25	8.19
19	4-chlorophenyl arsonic acid	2.33	2.21	3.33	8.25
20	4-methylphenyl arsonic acid	2.16	2.04	3.70	8.68
21	4-methoxyphenyl arsonic acid	2.14	2.00	3.79	8.93
22	4-aminophenyl arsonic acid	2.06	1.92	4.13	9.19
23	4-nitronaphthalen-1-yl-1-arsonic acid	2.40	2.39		7.87
24	4-nitrophenyl arsonic acid	2.52	2.42	2.90	7.80
25	3-acetylamino-4-hydroxyphenyl arsonic acid	2.19	2.31	3.78	7.9
26	4-hydroxy-3-nitrophenyl arsonic acid	2.43	2.32	3.46	
27	4-hydroxyphenyl arsonic acid	2.17	2.04	3.89	8.37
28	benzyl arsonic acid	2.14	2.14	3.81	8.49
29	butyl arsonic acid	2.10	2.10	4.23	8.91
30	ethyl arsonic acid	2.12	2.12	3.89	8.35
31	hexyl arsonic acid	2.09	2.09	4.16	9.19
32	methyl arsonic acid	2.16	2.16	3.41	8.18
33	pentyl arsonic acid	2.09	2.10	4.14	9.07
34	phenyl arsonic acid	2.21	2.09	3.47	8.48
35	propyl arsonic acid	2.11	2.11	4.21	9.09

For the correlation with the atomic charges on the carboxylate method by Monard et al.,^56^ all geometries were optimized at the M06-2X level of theory, using the SDD basis set for As and the 6-311G(d,p) basis for all remaining atoms (namely, C, H, and O), using the aforementioned suite of programs. Once again, all molecules were checked to ensure that there were no imaginary frequencies. Natural population analysis (NPA) was performed on the resulting structures to obtain the formal charges on the atoms of interest. The highest and average oxygen natural atomic charges of the conjugate arsonate oxygen atom fragment were compared with the experimental p*K*_a_ of the corresponding molecule. From these three, the average NPA oxygen charges yielded the best agreement with the experimental p*K*_a_ values and were thus used throughout the study.

A linear equation is obtained by a least-squares fit for the *Q* descriptor shown in eq 3, which is the average atomic charge of the arsonate oxygens. The predicted p*K*_a_’s are computed using eq 3 (i.e., by reporting average{*q*(O_1_), *q*(O_2_), *q*(O_3_),} of a given molecule into the parametrized equation).
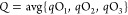
3

The third DFT approach developed by Lian et al.^57^ is based on the optimal description of the solute–solvent boundary, an essential component of continuum solvation models. To calculate the bulk electrostatic contribution for the default SMD model (described as SMDDefault), the solute–solvent boundary and cavity are optimized, and sSAS is used to construct the cavity in the SMD continuum model. This is known as SMDsSAS. The SCRF section of SMDsSAS allowed simultaneous tuning of the surface type and scaling factor options. The solvent radius, here taken as 1.385 times the radius of water, is used to expand the Coulomb radii of atoms to construct a more realistic cavity representing the molecule surrounded by the solvent. The scaling factor (0.4–0.8) is used to adjust the size of the SAS cavity, which is important for tuning solvation models and calculating the SASA of molecules. This way of using the approach helps for tuning the solvent to “see” the solute, impacting on the calculations such as solvation energies, binding free energies, and other properties where solute–solvent interactions play a key role. While the *V*_S,max_ and SAS approach was not effective for arsonic acids, we ML methods to overcome these limitations. Specifically, we used the SVM approach developed by Cortes and Vapnik^58^ with sparse generalization applicability and that is widely used in drug and material design.^59−61^ SVM allowed us to achieve better predictive accuracy for the p*K*_a_ values of arsonic acids. The ML approach was less affected by the unique complexities of the arsenic atom, including the delocalization of charge and solvation effects, and therefore provided a more reliable model for these compounds. The SVM, over the other algorithms, has an advantage in that it can be used for small data sets. All SVM calculations in this work were conducted by using AlvaModel.^62^ The test and train sets are selected randomly with a test:train ratio equal to 1:4. The algorithm selects the optimal hyperplane that maximizes the margin between the different class labels. This margin is defined by the vectors (support vectors) that are closest to the hyperplane, which ensure the robustness of the classification boundary.

## Results and Discussion

### *V*_S,max_-Based Calculation for p*K*_a_

The two highest maximum values of EPS around the acids are located just in front of the acidic hydrogen atoms. The highest maximum value was labeled as *V*_S,max1_, and the second highest maximum value was labeled as *V*_S,max2_, as shown in Table 1.

All four correlations between *V*_S,max1_, *V*_S,max2_, and p*K*_a1_ and p*K*_a2_ were evaluated after obtaining the fitted linear equation and their *r*^2^ correlation coefficients for each case. Next, these equations were used to predict the p*K*_a_ of the same acids. Then, compared to the predicted p*K*_a_ and experimental p*K*_a_, the absolute error and mean absolute error were calculated. Finally, a cross-validation test and graphical plot of residuals were carried out to prove the method’s robustness.

To determine what p*K*_a_ data correlated better with the maximum value of EPS, all possible correlations between them were explored and the results are summarized in Table 2. The second column corresponds to the experimental p*K*_a1_ value. In contrast, the third column is the calculated p*K*_a1_ obtained by using the *V*_S,max1_ and p*K*_a1_ correlation equation, the fourth column is the p*K*_a1_ calculated using the *V*_S,max2_ and p*K*_a1_ correlation equation, the fifth column is the experimental p*K*_a2_ value, the sixth column is the p*K*_a2_ calculated using the *V*_S,max1_ and p*K*_a2_ correlation equation, and the seventh is the p*K*_a2_ calculated using the *V*_S,max2_ and p*K*_a2_ correlation equation.

**Table 2:** Experimental p*K*_a1_ and p*K*_a2_ Values and Predicted p*K*_a_ Values of p*K*_a1cal_ and p*K*_a2cal_ for Each Correlation

no.	p*K*_a1_	p*K*_a1calc_	p*K*_a__1calc_	p*K*_a2_	p*K*_a2calc_	p*K*_a__2calc_
1	3.66	3.87	3.82	8.66	8.70	8.65
2	3.22			8.46	8.55	8.72
3	4.35	4.28	4.19	9.55	9.27	9.23
4	3.68	3.48	3.41	8.37	8.17	8.03
5	3.76	3.63	3.89	8.39	8.39	8.75
6	3.82	3.89	3.80	8.85	8.74	8.63
7	4.08	4.16	4.07	9.40	9.10	9.03
8	3.79	3.57	3.91	8.93	8.29	8.80
9	3.37	3.87	3.78	8.54	8.71	8.59
10	4.00	4.11	4.02	7.92	9.03	8.96
11	3.95	3.76	3.67	8.85	8.55	8.43
12	3.51	3.76	3.72	8.31	8.56	8.49
13	3.71	3.75	3.71	8.77	8.55	8.49
14	3.63	3.62	3.58	8.53	8.37	8.28
15	3.82	3.80	3.89	8.60	8.62	8.75
16	3.41	3.29	3.37	7.80	7.92	7.96
17	4.22	3.54	3.61	8.44	8.26	8.33
18	3.25	3.54	3.63	8.19	8.26	8.37
19	3.33	3.55	3.64	8.25	8.27	8.38
20	3.70	3.82	3.92	8.68	8.64	8.80
21	3.79	3.85	3.97	8.93	8.69	8.88
22	4.13	3.98	4.10	9.19	8.85	9.08
23				7.87	8.14	7.95
24	2.90	3.26	3.32	7.80	7.87	7.88
25	3.78	3.78	3.49	7.9	8.58	8.15
26	3.46	3.39	3.48			
27	3.89	3.80	3.91	8.37	8.62	8.78
28	3.81	3.85	3.76	8.49	8.68	8.56
29	4.23	3.91	3.82	8.91	8.76	8.65
30	3.89	3.88	3.79	8.35	8.72	8.60
31	4.16	3.92	3.83	9.19	8.78	8.67
32	3.41	3.81	3.72	8.18	8.63	8.50
33	4.14	3.92	3.82	9.07	8.78	8.66
34	3.47	3.74	3.83	8.48	8.53	8.67
35	4.21	3.90	3.81	9.09	8.75	8.64

The absolute error between the experimental and calculated p*K*_a_'s has values between 0.00 and 1.04, indicating that in some acids, the predicted value is equal to the experimental value, and in other cases, the calculated values move away up to 1.04 p*K*_a_ units with these correlations. The mean absolute error (MAE) tells us, on average, how far our calculated values are from the experimental values. MAE for the *V*_S,max1_ and p*K*_a1_ relationship is 0.18, and MAE for the *V*_S,max2_ and p*K*_a1_ relationship is 0.20; therefore, the value of *V*_S,max1_ correlated with p*K*_a1_ predicts better values for p*K*_a1_ than *V*_S,max2_ with p*K*_a__1_ correlation. In the same way, the MAE for *V*_S,max1_ and *V*_S,max2_ correlated with p*K*_a2_ is 0.25, meaning that both correlation equations predict the values of p*K*_a2_ with similar accuracy.

**Figure 2:**
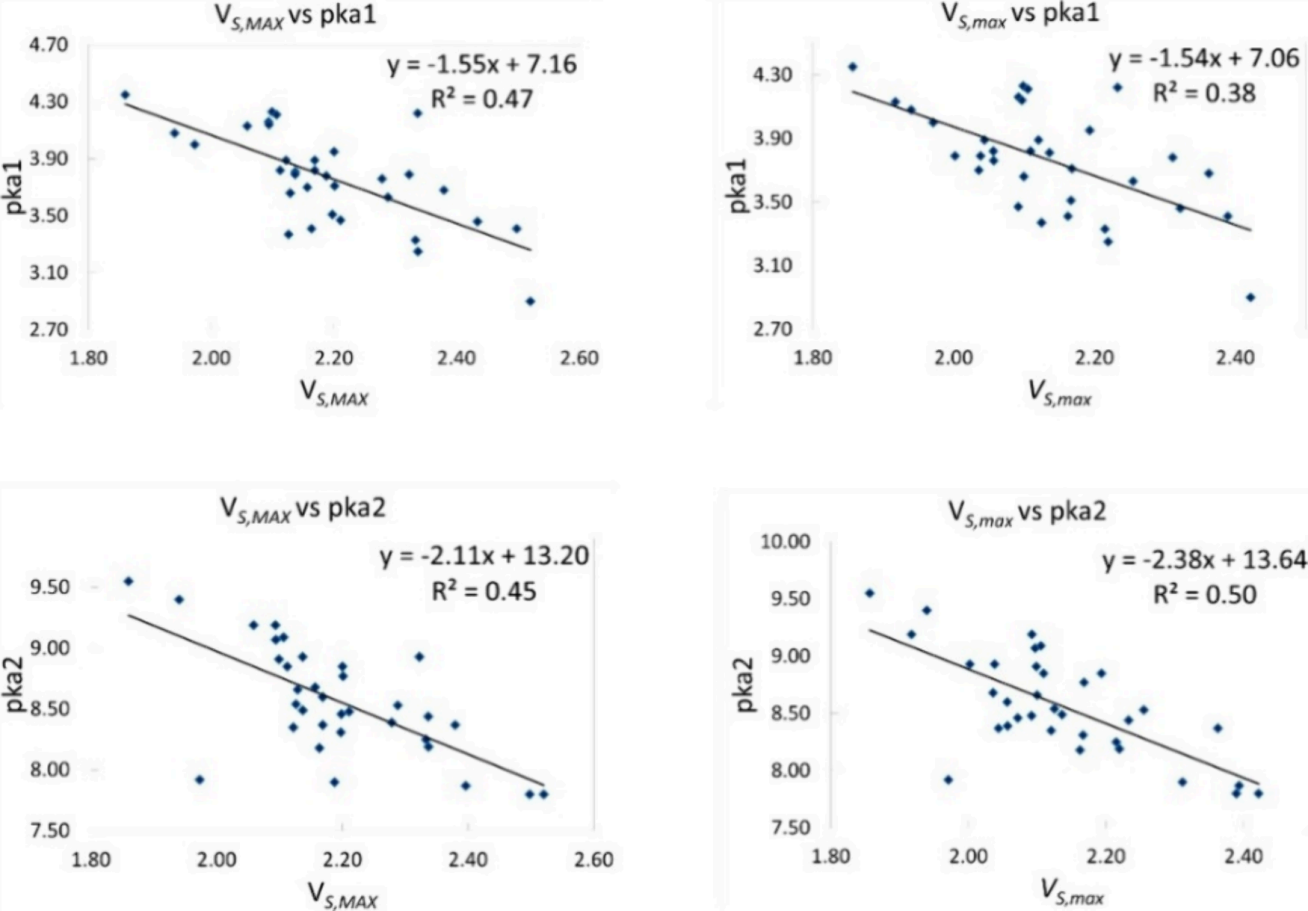
Correlation of *V*_S,max1_ (top row) and *V*_S,max2_ (bottom row) vs p*K*_a__1_ and p*K*_a__2_.

Figure 2 shows all linear correlations graphically with their respective fitted equations and correlation coefficient (*r*^2^) values. As previously mentioned, these equations are used to predict p*K*_a_, as shown in Table 2. The obtained correlation coefficient can be defined as moderate to low. The poor correlation implies that using *V*_S,max_ only is not an appropriate method to accurately predict the p*K*_a_ values for the explored set of acids. Nonlinear relationships might exist between the variables that the current linear model is unable to capture.

The residual plots were studied to determine the possible reason and solution for the low value of *R*^2^ (see Figure 2); in these graphs, the residuals or the error between the experimental and theoretical values were plotted. No trends or agglomerate data are needed in residual plots to support the hypothesis of a linear correlation between the data. Fortunately, the residual data are randomly dispersed in the plot, which means that the linear model is adequate for these data. Moreover, in the residuals, it is possible to appreciate what values have the greatest error and, in some cases, treat those data as an outlier and, because of that, delete them from the whole set. An additional statistical test to ensure that the data follow a linear correlation is the *F* test, which analyzes the fit applied to a set of data under the acceptance or rejection of the null or alternative hypothesis; in these cases, the calculated *F* is higher than the critical one, supporting once again that the data have a linear correlation, which is the alternative hypothesis.

**Figure 3:**
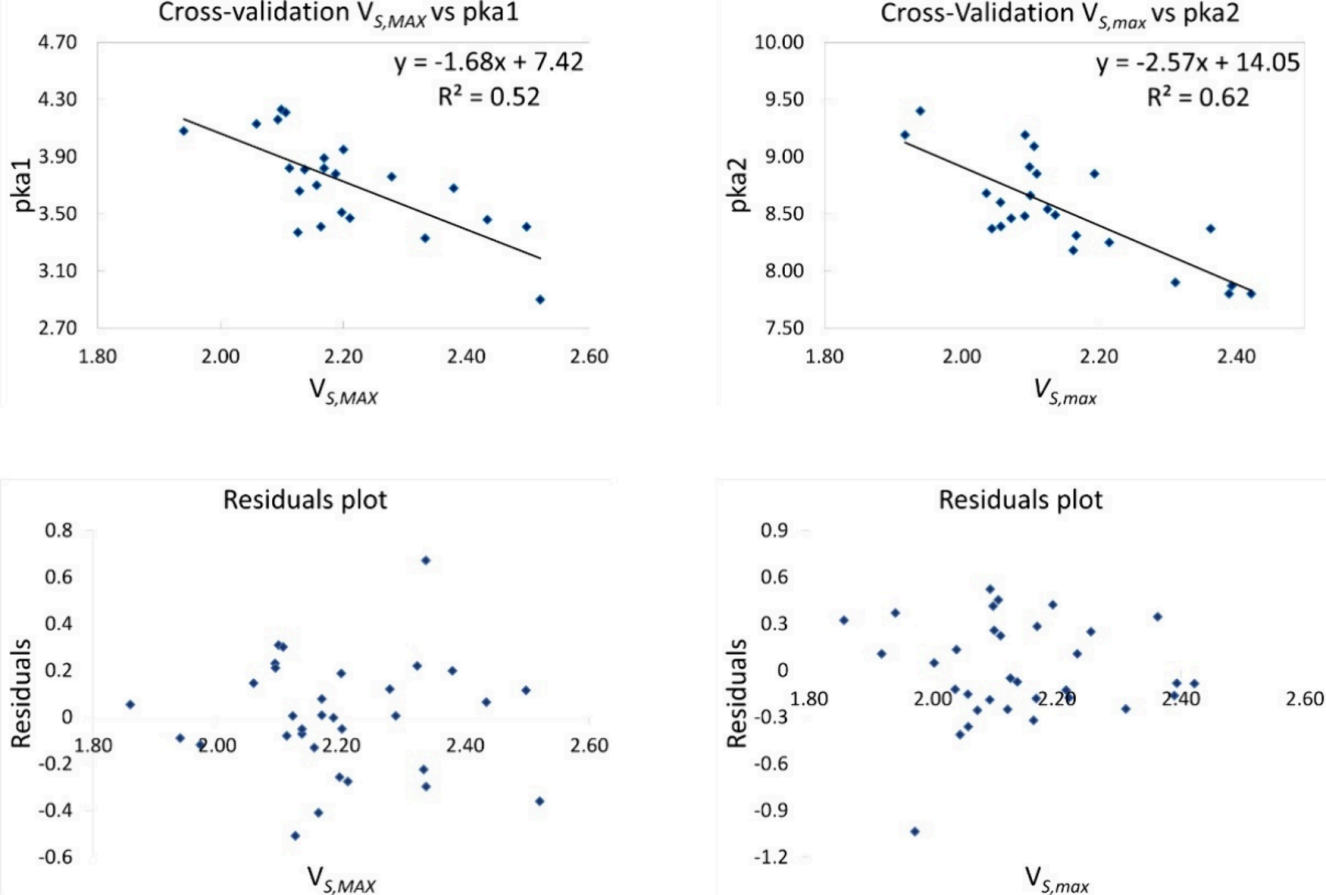
Residual and cross-validation plots.

However, it is insufficient to prove that the data follow a linear correlation to use them as a model to predict the values. The cross-validation test is very helpful in creating a robust predictive model (see Figure 3). The cross-validation method tells us that if we subtract randomly a small subset of the whole set and do the same procedure to predict the values; the new predicted values must have the same accuracy as the predicted values with the correlation obtained from all data. This result helps us to ensure that the predicted results are independent of the partition between the training and test data. Therefore, 10 of the 35 values were randomly eliminated. Next, the data were plotted, obtaining new correlation equations used to predict p*K*_a__1_ and p*K*_a__2_. Similar results were found using the complete set of data and the data selected on the cross-validations test. The *R*^2^ value, in this case, improves, but the predicted values have the same accuracy.

### Quantum Chemical Calculation of p*K*_a_ by sSAS: Assessing the Effect of the Cavity Scaling Factor (α) on the p*K*_a_ Values

In the following section, the two approaches were used for p*K*_a_ prediction using the standard SMD model and the SMD model but coupled with a scaled solvent-accessible surface to define the solute–solvent boundary more precisely. The chosen approach for p*K*_a_ calculations with the application of a continuum solvent directly included eliminating the need for thermodynamic cycle calculations. MAE was employed to measure the predictive accuracy for each method, specifically comparing the impact of these approaches on p*K*_a_ calculations for arsenic atoms and the corresponding cavity scaling values. Drawing inspiration from the work of Smith et al.,^63^ who demonstrated improved p*K*_a_ predictions for functional groups like carboxylic acids, amines, and thiols by tuning the sSAS in the SMD, a similar optimization process was conducted. We performed an optimization search for the scaling factor α within the range of 0.4–0.8 to identify the value that minimized the MAE. This approach allowed us to refine the solute–solvent boundary representation, enhancing the model’s accuracy by accounting for solvation effects more realistically in the case of arsonic acids. The resulting optimized α represents a balance between the solute cavity size and solvation interactions, critical for reliable p*K*_a_ predictions in complex molecular systems.

The p*K*_a_ was calculated with the scaled value of α and without correction to the cavity size. The calculation of p*K*_a_ values was based on the Arrhenius equation .

Unfortunately, the prediction rate is unacceptable for either of the chosen approaches based on SMD. The obtained p*K*_a_ for the arsonic acid data vs experimentally measured p*K*_a_ values are presented in the Supporting Information. The SMD variants without the effect of the scaling factor of this data set were better, but they were far from having a reasonable prediction rate.

The p*K*_a_'s calculated with different SMD variants show no correlation with the corresponding experimental values, with an *R*^2^ value of 0.45 for the SMD default, which was the best within the two methods. However, for the set of arsonic acids, the obtained results are insufficient to use the two approaches compared with the published results for some other acids.

Despite the effectiveness of *V*_S,max_ and SAS for many organic acids, the approach faced limitations in the case of arsonic acids. The unique electronic structure of the arsenic atom, coupled with the presence of multiple acidic sites, introduced complexities that were not adequately captured by the *V*_S,max_ descriptor alone. Specifically, the delocalization of charge across the arsonate group and the influence of arsenic’s d-orbitals created discrepancies between the predicted and experimental p*K*_a_ values. Additionally, the solvation effects modeled through SAS were less accurate due to the distinct solvation behavior of arsonic acids compared with simpler carboxylic acids. As a result, although the methodology provided reasonable estimates, it was insufficient to achieve the desired level of accuracy for these compounds.

### Atomic Charge-Based Mode for the p*K*_a_ Calculations

The next approach was to link partial atomic charges to p*K*_a_ experimental data. Monard et al.^56^ reported a benchmarking study based on NPA charges computed by using the CPCM solvation model with the B3LYP/3-21G level. This resulted in the most accurate combination for reproducing the experimental p*K*_a_ values for alcohols, thiols, and amino acids. While other charge models, such as Mulliken, Löwdin, and AIM charges, can also be used to predict p*K*_a_, the NPA charge scheme consistently outperforms other methods.

Our study utilized the NPA charges on the oxygen to estimate p*K*_a_ values, a method that demonstrated very high correlation coefficients. The NPA charges, a key aspect of our research, play a crucial role in understanding the acidity of the compounds and their potential environmental impact.

The NPA was used to calculate atomic charges. The methodology for revealing the linearity of the relationship between experimental p*K*_a_’s and atomic charges was inspired by the work of Ugur et al.^56^

Using the SMD implicit solvent model, the average charge on the oxygen of each arsonic fragment was computed with NPA at M06-2X/SDD (for the arsenic atom).

According to Ugur et al.,^56^ the negative charge of carboxylate can be shared between two oxygen atoms and two carbon atoms, as opposed to alcohols and thiols. The atomic charges for this fragment can be extracted in various ways and then compared with experimental p*K*_a_ values using eq 3.

**Figure 4:**
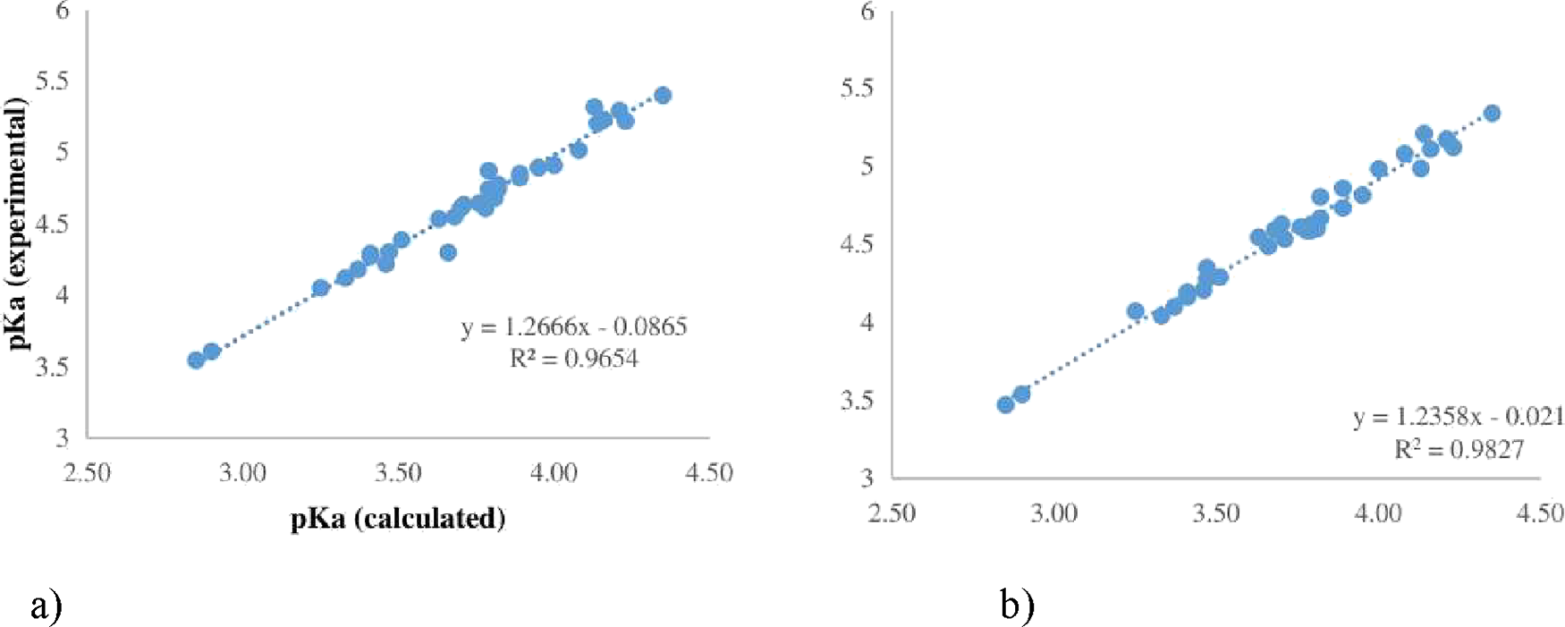
Linear correlation of experimental p*K*_a_’s and values calculated using M06-2X and 6-311G(d,p) with SMD based on (a) average oxygen charges and (b) oxygen charges for the monoprotonated form (anion).

Our present protocol for obtaining accurate and fast p*K*_a_ predictions for a limited set of arsonic acids unveils a new pattern in the charge extraction scheme, setting our study apart from another available research. The linear regression was evaluated between the experimental p*K*_a_’s and the average NPA atomic charges on the ionizable OH groups presented in Figure 4. The best combination of DFT functionals and basis sets is M06-2X/SDD with the SMD default model. The next goal would be to transfer the suggested extracted scheme of charge protocol to a set of tricarboxylic acids by calculating the average atomic charge of the carboxylate that forms into tricarboxylic acids, such as hemimellitic acid (1,2,3-benzene tricarboxylic acid), trimellitic acid (1,2,4-benzene tricarboxylic acid), and trimesic acid (1,3,5-benzene tricarboxylic acid). These tricarboxylic acids can act as environmental pollutants due to their acidic nature and potential toxicity. The p*K*_a_ values for these tricarboxylic acids vary depending on the positions of the carboxyl groups on the benzene ring. The other test group will be perfluorooctanoic acid, which has a significantly different p*K*_a_ value on the air–water surface compared to the reported bulk p*K*_a_ values. Focusing our interest in accurate p*K*_a_ values of PFAS is essential for understanding and modeling their environmental fate and transport as p*K*_a_ determines the speciation and behavior of these persistent pollutants.

### Machine Learning Approach for p*K*_a_ Prediction

**Figure 5:**
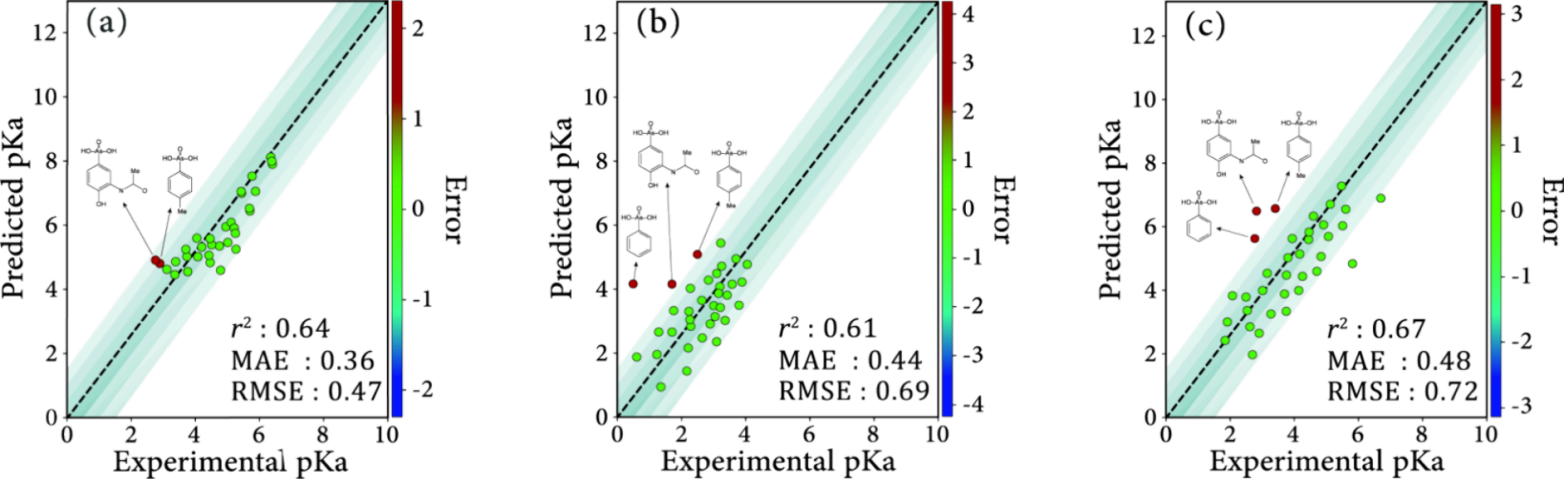
Comparison between predicted and experimental p*K*_a_ values based on the SVM model for the selected combination of the descriptors. Model a: simple with fewer descriptors, primarily focused on solubility and structural connectivity. Model b: enhanced with more descriptors related to charge distribution, lipophilicity, and molecular structure, improving accuracy. Model c: the most comprehensive model, capturing a wide range of molecular properties like polar surface area, volume, charge, and connectivity, provides the highest accuracy for p*K*_a_ prediction.

The data sets containing the experimental values of p*K*_a__1_ were modeled using the SVM algorithm, with the results shown in Figure 5. For the deprotonated form of the arsonic acids, a set of molecular descriptors was generated from their three-dimensional conformations using Alvadesc.^62^ A comprehensive set of molecular descriptors was initially calculated for the first ionization form of the arsonic acids, starting with a descriptor space of 4000 descriptors categorized into 22 classes. These classes included constitutional descriptors, topological descriptors, walk and path counts, connectivity indices, information indices, 2D autocorrelations, edge adjacency indices, Burden eigenvalues, topological charge indices, eigenvalue-based indices, Randic molecular profiles, geometrical descriptors, RDF descriptors, 3D-MoRSE descriptors, WHIM descriptors, GETAWAY descriptors, functional group counts, Ghose-Crippen atom-centered fragments, charge descriptors, molecular properties, 2D binary fingerprints, and 2D frequency.

To refine the model, 94 descriptors were preselected. The selection process involved using a genetic algorithm–multilinear regression approach to identify features with the highest relevance. The descriptors are presented in the Supporting Information for each of the tested models. These descriptors were selected based on their contribution to the prediction accuracy, and they range from 1 to 20 in number.

The SVM model was then employed to predict the p*K*_a_ values using the optimal combination of selected descriptors listed in Table S2 of the Supporting Information. The predicted p*K*_a_ values obtained from this machine learning method are plotted against the experimental values in Figure 5, and each plot represents an obtained mode on different descriptor sets. The selected descriptors for each model indicate the different molecular features that were prioritized to optimize p*K*_a_ prediction accuracy. Each descriptor provides unique information about the molecular structure, polarity, electronic properties, or solubility, contributing to the model’s ability to predict p*K*_a_ values effectively.

The selected descriptors in each model progressively add complexity, with model 3 offering the most complete set for accurate p*K*_a_ prediction by covering essential molecular properties that affect proton dissociation.

The model demonstrates a good predictive capability, as indicated by the *R*^2^ values for each combination of descriptors. In the plots, outliers are highlighted with red dots, representing molecules that deviate from the main trend. These outliers are identified as cases where the predictions are biased relative to the experimental values.

These descriptors are related to the p*K*_a_ values in the following manner. The McGowan volume (*V*_x_) is one of the key parameters used to evaluate and predict the p*K*_a_ values of the compounds. It is considered an Abraham descriptor, a type of molecular descriptor commonly used in quantitative structure–activity relationship (QSAR) modeling. For instance, studies have successfully employed the McGowan volume alongside other descriptors, such as log *P*, to model the p*K*_a_ values of chlorinated phenols. The McGowan volume is particularly effective as a molecular descriptor for predicting p*K*_a_ values as it reflects the steric effects and molecular size that influence the ionization process.

In our study, the *V*_x_ descriptor was used to accurately predict p*K*_a_ values based on leverage models. This approach highlights the importance of considering both the molecular volume and its relationship to the ionization energy when developing predictive models for p*K*_a_. By incorporating *V*_x_ into the SVM model, we can achieve precise predictions of p*K*_a_ values, demonstrating the descriptor’s relevance and utility in quantitative structure–property relationship (QSPR) modeling.

The McGowan volume provides a compact and easy-to-interpret representation of molecular size and shape, which can be helpful for p*K*_a_ prediction.^64^ Connectivity indices (X3Av), also known as branching indices, are a class of topological descriptors that capture essential information about the molecular structure and connectivity of atoms within a molecule. These indices quantify the degree of branching in a molecular structure, providing insights into how atoms are connected and how this connectivity influences the molecule’s overall properties. In the context of p*K*_a_ prediction, connectivity indices like X3Av are particularly valuable because they reflect the structural complexity and branching patterns that can affect the distribution of electron density and, consequently, the molecule’s ionization potential. By incorporating these topological descriptors into our predictive models, we can gain a more comprehensive understanding of how molecular connectivity influences p*K*_a_ values, leading to more accurate predictions.^65−67^ X3Av is a numerical descriptor that encapsulates information about the molecular graph (the way atoms are connected) of a compound. This index helps in predicting the physicochemical properties, biological activities, and chemical reactivity of molecules based on their structure. These indices quantify the degree of branching and connectivity in a molecule, which can be relevant for predicting properties like p*K*_a_. The used 2D autocorrelations (ATS1m) as molecular descriptors are one of the types of molecular descriptors that can capture information about the distribution of specific properties, (e.g., atomic properties and bond properties,) along the 2D molecular structure. This suggests that 2D autocorrelation descriptors can provide relevant information for predicting the p*K*_a_ values of molecules as they capture structural features that influence the acid–base behavior. The 2D autocorrelations and other molecular descriptors are commonly used as inputs for developing QSAR and QSPR models to predict the p*K*_a_ and other properties.

TPSA, or topological polar surface area of a molecule, is a critical measure defined as the total surface area of all polar atoms within a molecule·^68^ The polar surface area has been used in medicinal chemistry to optimize a drug’s potential to permeate cells,^69,70^ and it is considered a main descriptor to evaluate the blood–brain barrier penetration.^71^ According to Lipinski’s rule of five, an orally active drug typically has a TPSA less than 140 Å^2^ and a p*K*_a_ value that ensures that the molecule is not too ionized at physiological pH. Adjusting TPSA and p*K*_a_ helps achieve a balance between solubility and permeability, enhancing oral bioavailability. Compounds with a TPSA less than 90 Å^2^ are more likely to cross the blood–brain barrier. Incorporating p*K*_a_ values into predictive models helps to determine the ionization state of these compounds at physiological pH, which significantly affects their ability to permeate biological membranes, including the blood–brain barrier. The ionization state influences a compound’s lipophilicity, solubility, and overall pharmacokinetic profile, making p*K*_a_ a crucial parameter in drug design, particularly for targeting neurological conditions. By integrating both TPSA and p*K*_a_ values, researchers can more accurately predict the likelihood of a compound reaching its target site within the brain, thus optimizing the drug efficacy and safety. Furthermore, the MLOGP^72^ descriptor plays a significant role in QSAR models, where it is frequently employed to predict the permeability of compounds across the blood–brain barrier. This is particularly important for developing drugs to treat neurological conditions as it helps researchers predict how effectively a molecule can deliver therapeutic effects to the brain. By using MLOGP and other molecular descriptors, scientists can better understand the pharmacokinetic properties of new drug candidates, leading to more effective and targeted drug therapies. The other group of descriptors is atom-centered fragments (C-002), which are a type of molecular descriptor that can capture information about the environment surrounding each atom in a molecule.^73^ The combination of various types of molecular descriptors, including MLOGP, atom-centered fragments, connectivity indices, and 2D autocorrelations, enables the development of robust predictive models for p*K*_a_ values. These descriptors collectively provide comprehensive information about the chemical environment and structural features of molecules, which are essential for accurate property predictions.

## Conclusions

Four computational models were contrasted to assess p*K*_a_'s of 35 arsonic acids quickly; three were based on DFT calculations, and the fourth was based on an SVM. Accurate prediction of these values for arsonic acid derivatives is essential in planning their extraction strategies. However, this has proven to be a more elusive task than organic molecules, such as carboxylic acids or thiols. Contrary to our initial expectations, neither ML nor correlation to *V*_S,max_ calculations provided acceptable MAE values, and instead, the method proposed by Smith et al. for the scaled SAS SMD solvation model yields the best predictions for the present family of arsonic acids. While DFT models provide highly detailed and accurate predictions, the SVM model offers a potentially faster and more efficient alternative provided that it is well-trained on relevant data. The comparison aimed to identify the best method for reliable and expedient predictions.

## Data Availability

The data files with generated descriptors, obtained charges, and *xyz* coordinates for the arsonic acids used in this study are available at https://github.com/mici345/pKa_input_files.

## Supporting Information Available

Table S1: calculated atomic charges for each atom under study, details of charge per atom for each molecule, calculated using NPA, and experimental p*K*_a__1_ values; Table S2: information about the model’s set of descriptors that was tailored to capture different aspects of molecular behavior, with increasing complexity and specificity, leading to improved predictive accuracy as reflected by the presented *R*_adj_^2^ values; table with the α scaling factors (PDF)
