# Supplementary material for: Comparative Analysis of pKa Predictions for Arsonic Acids Using Density Functional Theory-Based and Machine Learning Approaches
Source: ACS Omega. 2025 Jan 16;10(3):3128–40. doi: 10.1021/acsomega.4c10413 (PMC11780423; doi:10.1021/acsomega.4c10413)
Supplement: Supplementary file 1 — ao4c10413_si_001.pdf [file ao4c10413_si_001.pdf]

Supporting Information

Comparative Analysis of pKa Predictions for Arsonic Acids Using DFT-Based and Machine Learning Approaches

Miroslava Nedyalkova[‡,§,±]*, *Diana Heredia*[||], *and Joaquín Barroso-Flores*[†,¶] *and Marco Lattuada*[§]*

[‡]*Swiss National Center for Competence in Research (NCCR) Bio-inspired Materials, University of Fribourg, Chemin des Verdiers 4, CH-1700 Fribourg, Switzerland*

[§]*Department of Chemistry, University of Fribourg, Chemin du Musée 9, Fribourg 1700, Switzerland*

[±]*Department of Inorganic Chemistry, Faculty of Chemistry and Pharmacy, University of Sofia 'St. Kl. Ohridski', Sofia, Bulgaria.*

[||]*School of Chemical Sciences and Engineering, Yachay Tech University, 100119 Urcuquí, Ecuador*

[†]*Centro Conjunto de Investigación en Química Sustentable UAEM-UNAM, Carretera Toluca-Atlacomulco Km 14.5, Unidad San Cayetano, Toluca, Estado de México, 50200. México*

[¶]*Instituto de Química, Universidad Nacional Autónoma de México. Circuito Exterior S/N Ciudad Universitaria, Alcaldía Coyoacán, Ciudad de México, CP 05410 México*

E-mail: miroslava.nedyalkova@unifr.ch

Table S1: NPA (Natural Population Analysis) calculated the charge for each atom and the experimental pKa_1 values.

**Name**	**As**	**O1**	**O2**	**O3**	**pKa_1**
1-Naphthylarsonic	2,53954	-1,23788	-1,21722	-1,09198	8,66
2_4-Dimethoxyphenylarsonic	2,54865	-1,23127	-1,22834	-1,09185	9,55
2-Aminophenylarsonic	2,54823	-1,25176	-1,21106	-1,08915	8,93
2-Chloroethylarsonic	2,52406	-1,23632	-1,22894	-1,10787	8,37
2-Chloropropylarsonic	2,5249	-1,23577	-1,22744	-1,10756	8,39
2-Hydroxyphenylarsonic	2,54766	-1,22764	-1,22593	-1,09128	7,92
2-Methoxyphenylarsonic	2,54713	-1,22935	-1,22627	-1,09068	9,40
2-Methylphenylarsonic	2,54016	-1,24168	-1,21833	-1,0991	8,85
2-Naphthylarsonic	2,54257	-1,24104	-1,21743	-1,09314	8,46
2-Nitrophenylarsonic	2,55223	-1,21944	-1,21402	-1,08434	8,54
3-Acetylamino-4-hydroxyphenylarsonic	2,54423	-1,23927	-1,21971	-1,09327	7,90
3-Chlorobutylarsonic	2,53099	-1,25034	-1,22322	-1,10582	8,85
3-Chlorohexyl-1-arsonic	2,53276	-1,24931	-1,22324	-1,10585	8,31
3-Chloropentyl-1-arsonic	2,53238	-1,24954	-1,22335	-1,10586	8,77
3-Chloropropylarsonic	2,5295	-1,25044	-1,22286	-1,1057	8,53

3-Methylphenylarsonic	2,541	-1,24342	-1,22176	-1,09444	8,60
3-Nitrophenylarsonic	2,5442	-1,23753	-1,20844	-1,09064	7,80
4-Aminonaphthalen-1-yl-1-arsonic	2,54025	-1,24046	-1,21958	-1,09364	8,67
4-Aminophenylarsonic	2,54254	-1,24686	-1,22397	-1,09521	9,19
4-Arsonobenzoic	2,54015	-1,23782	-1,21469	-1,09182	4,22
4-Bromophenylarsonic	2,5426	-1,2394	-1,21711	-1,09266	8,19
4-Chlorophenylarsonic	2,54254	-1,24003	-1,21775	-1,09295	8,25
4-Hydroxyphenylarsonic	2,54282	-1,24581	-1,22159	-1,09487	8,37
4-Methoxyphenylarsonic	2,54277	-1,24568	-1,22212	-1,09501	8,93
4-Methylphenylarsonic	2,54097	-1,24431	-1,22176	-1,0948	8,68
4-Nitronaphthalen-1-yl-1-arsonic	2,53939	-1,22842	-1,20887	-1,08851	7,87
4-Nitrophenylarsonic	2,54158	-1,23397	-1,21147	-1,09045	7,80
Benzylarsonic	2,53164	-1,24735	-1,22023	-1,09931	8,49
Butylarsonic	2,52512	-1,25725	-1,22935	-1,10764	8,91
Ethylarsonic	2,52008	-1,25888	-1,23095	-1,10878	8,35
Hexylarsonic	2,52527	-1,25707	-1,22899	-1,10768	9,19
Methylarsonic	2,50089	-1,24047	-1,23381	-1,01799	8,18
Pentylarsonic	2,52509	-1,25715	-1,22911	-1,10769	9,07

Phenylarsonic	2,5406	-1,24354	-1,221	-1,09446	8,48
Propylarsonic	2,52435	-1,25763	-1,22969	-1,10781	9,09

Table S2: Selected models based on high scored set of descriptors. The improved predictive accuracy as was represented by the calculated R^2_{adj} values.

**Model**	**Selected descriptors**
Model 1	ATS1m, ESOL, X4Av
Model 2	MAXDP, TIE, C-002, ALOGP, X1Av, nDB
Model 3	TPSA(NO), MAXDP, Ds, Vx, TIE, MLOGP, RNCG, X3Av

**Model**	**Training Set MAE**	**Estimated Test Set MAE**	**Estimated Test Set RMSE**
Model 1	0.36	0.42	0.52
Model 2	0.44	0.50	0.76
Model 3	0.48	0.50	0.79

Model (1) has the smallest MAE difference between the training and test sets, suggesting good generalization with minimal overfitting.

Model (2) shows a moderate increase in MAE on the test set, indicating reasonable but slightly lower generalizability.

Model (3) has the largest increase in MAE from training to test, suggesting the highest level of overfitting and the least generalizable performance among the three models.

Model 1: Basic Molecular Properties

Descriptors: ATS1m, ESOL, X4Av

ATS1m (Atomic Topological State Index of order 1m): Reflects molecular size or volume through a topological approach, contributing to understanding how steric factors affect the property being modeled.

ESOL (Estimated Solubility): A descriptor that estimates aqueous solubility, influencing how a molecule interacts with its environment, which is crucial for processes like absorption and distribution.

X4Av (Average of Chi-4 index): Provides information about the connectivity of atoms within a molecule, impacting molecular stability and reactivity.

Model 1 integrates basic molecular descriptors that capture size, solubility, and connectivity, making it suitable for general predictions where the primary interactions are steric, and solvation driven.

Model 2: Enhanced Chemical Interaction and Partitioning

Descriptions : MAXDP, TIE, C-002, ALOGP, X1Av

MAXDP (Maximum Diameter of Perpendiculars): Captures the largest cross-sectional diameter, which can influence molecular packing and transport properties.

TIE (Topological Index E): A measure of molecular branching, affecting how molecules fit together in a structured environment.

C-002: A specialized descriptor likely related to electronic or steric properties specific to the chemical family under study.

ALOGP (LogP): A measure of lipophilicity, essential for understanding solubility, permeability, and absorption.

X1Av (Average of Chi-1 index): Measures the connectivity of first-order neighbors in a molecule, impacting its chemical reactivity and biological activity.

Model 2 incorporates descriptors that refine predictions by considering molecular geometry, lipophilicity, and branching, which are critical for understanding how molecules interact in biological systems and environments.

Model 3 : Comprehensive Interaction and Environment Adaptation- R^2adj

Descriptors : TPSA, MAXDP, Ds, Vx, TIE, MLOGP, RNCG, X3Av

TPSA (Topological Polar Surface Area): Influences molecular interactions with polar environments, important for drug transport and enzyme interaction.

MAXDP, TIE, Vx (Molecular Volume): These descriptors, including volume and shape, are crucial for fitting into binding sites or transport channels.

Ds (Density): Affects solvation and settling behavior in different media.

MLOGP (Modified LogP): An enhanced logP calculation that better accounts for molecular partitioning in biological systems.

RNCG (Relative Negative Charge): Reflects the electron distribution and its impact on molecular polarity and reactivity.

X3Av (Average of Chi-3 index): Measures higher-order connectivity, offering insights into the complex binding or interaction capabilities.

Model 3 is the most comprehensive, utilizing a wide array of descriptors that account for molecular interaction with environments, biological activity potential, and transport properties, making it highly effective for detailed and specific predictive tasks.
